# Next-Generation Biomarkers for Cholangiocarcinoma

**DOI:** 10.3390/cancers13133222

**Published:** 2021-06-28

**Authors:** Pedro M. Rodrigues, Arndt Vogel, Marco Arrese, Domingo C. Balderramo, Juan W. Valle, Jesus M. Banales

**Affiliations:** 1 Department of Liver and Gastrointestinal Diseases, Biodonostia Health Research Institute, Donostia University Hospital, University of the Basque Country (UPV/EHU), 20014 San Sebastian, Spain; 2 National Institute for the Study of Liver and Gastrointestinal Diseases (CIBERehd, “Instituto de Salud Carlos III”), 28220 Madrid, Spain; 3 Department of Gastroenterology, Hepatology & Endocrinology, Hannover Medical School, 30625 Hannover, Germany; vogel.arndt@mh-hannover.de; 4 Department of Gastroenterology, Faculty of Medicine, Pontificia Universidad Católica de Chile, Santiago 8330077, Chile; marrese@uc.cl; 5 Department of Gastroenterology, Hospital Privado Centro Médico de Córdoba, Instituto Universitario de Ciencias Biomédicas de Córdoba, Córdoba 5000, Argentina; dbalderramo@hospitalprivadosa.com.ar; 6 Division of Cancer Sciences, University of Manchester, Manchester M13 9PL, UK; juan.valle@nhs.net; 7 Department of Medical Oncology, The Christie NHS Foundation Trust, Manchester M20 4BX, UK; 8 Department of Biochemistry and Genetics, School of Sciences, University of Navarra, 31008 Pamplona, Spain; 9 IKERBASQUE, Basque Foundation for Science, 48009 Bilbao, Spain

**Keywords:** cholangiocarcinoma, diagnosis, prognosis, biomarkers, omics

## Abstract

**Simple Summary:**

Early and non-invasive diagnosis of cholangiocarcinoma (CCA) is still challenging, thus largely contributing to the increased mortality rates observed worldwide. Consequently, several efforts have been made in order to report novel biomarkers for CCA, that would aid on diagnosis and also to predict prognosis and therapy response. We herein aim to provide an in-depth and critical revision on the next-generation biomarkers for CCA that have been recently proposed.

**Abstract:**

The increasing mortality rates of cholangiocarcinoma (CCA) registered during the last decades are, at least in part, a result of the lack of accurate non-invasive biomarkers for early disease diagnosis, making the identification of patients who might benefit from potentially curative approaches (i.e., surgery) extremely challenging. The obscure CCA pathogenesis and associated etiological factors, as well as the lack of symptoms in patients with early tumor stages, highly compromises CCA identification and to predict tumor development in at-risk populations. Currently, CCA diagnosis is accomplished by the combination of clinical/biochemical features, radiological imaging and non-specific serum tumor biomarkers, although a tumor biopsy is still needed to confirm disease diagnosis. Furthermore, prognostic and predictive biomarkers are still lacking and urgently needed. During the recent years, high-throughput omics-based approaches have identified novel circulating biomarkers (diagnostic and prognostic) that might be included in large, international validation studies in the near future. In this review, we summarize and discuss the most recent advances in the field of biomarker discovery in CCA, providing new insights and future research directions.

## 1. Introduction

Cholangiocarcinomas (CCAs) encompasse a group of highly aggressive biliary tract tumors with elusive etiopathogenesis that may arise throughout the biliary tree. They currently represent the second most common primary liver cancer (15%) after hepatocellular carcinoma (HCC), contributing approximately to 2% of cancer-related deaths yearly [1,2]. Although still being considered a rare cancer, CCA incidence (0.3–6 per 100,000 inhabitants yearly) has been increasing worldwide in the past decades, being particularly evident in specific regions of Southeast Asia (South Korea, China and Thailand), in which incidence rates are high (>6 per 100,000 inhabitants per year) [1]. CCAs are sub-classified according to their anatomical origin into intrahepatic (iCCA), perihilar (pCCA) or distal (dCCA) and were shown to display differences in etiopathogenesis, genetics, risk factors, clinicopathological features, management and outcome. Furthermore, pCCA and dCCA have been often grouped together under the collective term “extrahepatic” CCA (eCCA), but the use of this term is now strongly discouraged [1,2,3]. Several risk factors, such as age, benign inflammatory diseases (primary sclerosing cholangitis (PSC) and hepatolithiasis), liver cirrhosis, infectious agents (liver flukes (*Opisthorchis viverrini* and *Clonorchis sinensis*) and viral hepatitis), metabolic factors (obesity, diabetes and non-alcoholic fatty liver disease (NAFLD)), drugs/toxins (alcohol consumption, smoking, thorotrast, nitrosamines, asbestos, oral contraceptive pill, etc.) and congenital disorders (choledochal cysts, Caroli’s disease, congenital hepatic fibrosis) are known to predispose to CCA development; however, the great majority of tumors develop sporadically without a known underlying disease [1,4].

CCAs are frequently asymptomatic in early stages, particularly when the primary lesion does not cause biliary obstruction (as in iCCA); for pCCA and dCCA, diagnosis is usually made when symptoms secondary to tumor-related biliary obstruction and jaundice are evident [5]. The silent presentation and evolution of these tumors contribute to late diagnosis with surgery being possible in approximately 20% of patients [6]. This, combined with high tumor recurrence after surgery, results in dismal prognosis and high mortality rates of patients with CCA. In fact, despite improvements in diagnosis, management and treatment during the last years, the sensitivity and specificity of the current diagnostic tools is still limited and patients’ prognosis has not significantly improved, resulting in unsatisfactory 5-year survival rates (7–20%) [7,8,9,10]. Furthermore, mortality rates have been increasing globally in the last 2 decades [7,8,9,10,11], positioning CCA as an emerging health problem worldwide. Therefore, biomarker discovery is pivotal to improve patients’ welfare and outcome. Extensive research has been made in recent years to identify new biomarkers that contribute to the understanding of disease pathogenesis, aid in diagnosis, and also assist in predicting prognosis and therapeutic responses. In this review, we summarize the main findings regarding the potential next-generation biomarkers and biomarker discovery in CCA.

## 2. Biomarkers in the World of Clinical Needs

Accurate tumor biomarkers should help in the diagnosis of early-stage disease; should be prognostic (either for survival and/or potential tumor recurrence after surgery); or should help to guide therapeutic decisions (e.g., surgical, locoregional, systemic and targeted therapies). The ideal biomarker for CCA (and for other types of cancers) should be easily detectable (preferentially in a non-invasive manner), widely available and should be highly sensitive and specific, thus allowing the early diagnosis of the disease, when curative treatment can be applied. In addition, it should also allow the differential diagnosis of CCA and other malignant and/or benign diseases (such as HCC and PSC, respectively, among others). According to their usefulness, biomarkers might have a diagnostic, prognostic, predictive (for either therapy response and/or toxicity) and/or pharmacodynamic capacities, with some biomarkers possessing eventually more than one of these features. So far, although a lot of effort has been made in the field, we are still lacking accurate diagnostic, prognostic and predictive biomarkers for CCA.

### 2.1. Diagnosis of CCA

The diagnosis of CCA is usually achieved by combining clinical, radiological, serological and histological information [12,13]. Combining distinct approaches usually increase the sensitivity and specificity in CCA diagnosis, ensuring a more reliable result.

Cross-sectional imaging is an indispensable tool for CCA diagnosis. According to the subtype of CCA, distinct imaging techniques might be used [13,14]. Among all, computed tomography (CT) and magnetic resonance cholangiopancreatography (MRCP) are the better diagnostic modalities while functional imaging with positron emission tomography (PET) recently incorporated as the standard of care for disease staging and to identify tumor recurrence [15]. In patients with bile duct strictures suggestive of CCA, endoscopic retrograde cholangiopancreatography (ERCP) is indicated to assess and sample the biliary tree via brush cytology and/or endoscopic biopsy [16]. The main limitation of biliary cytology is the limited sensitivity (~45%) [16]; however, combining cytology with fluorescence in situ hybridization analysis increases the sensitivity of endoscopic cytology, although this approach is still not used routinely [17].

New technologies related to ERCP have also been studied. Single operator cholangioscopy (SOC) allows direct evaluation of biliary lesions and targeted biopsies. Sensitivity and specificity of SOC-targeted biopsies is 85 and 100%, respectively [18]. Microscopic evaluation of the biliary epithelium is also possible using probe-based confocal laser endomicroscopy (CLE). A prospective multicenter study that evaluated indeterminate biliary strictures demonstrated lower sensitivity and specificity compared to SOC [19]. Nevertheless, availability, cost, and standardizing training currently limit the global application of these techniques as routine procedures [20,21].

Histological analysis is required to establish the diagnosis. In addition, histopathological evaluation provides valuable information regarding tumor type and stage, aiding in the clinical management of patients [22]. However, sample collection for histological confirmation is sometimes complicated and not recommended in specific circumstances (for example, when considering transplantation for patients with pCCA) [23] due to tumor location and the increased risk of peritoneal seeding. Due to a lack of symptoms in early disease and not established screening programs, many patients with CCA are diagnosed with advanced-stage disease (~70%), limiting the possibility for curative treatment [12]. For this reason, the role of biomarkers to aid early diagnosis is of pivotal importance and has been under the spotlight in the past decade. Particularly, identifying biomarkers to predict CCA development in high-risk populations, such as patients with PSC and patients with choledochal cysts using samples that require non-invasive procedures to be obtained (e.g., serum, saliva and urine) is urgently necessary [12].

The serum tumor markers carbohydrate antigen (CA) 19-9 and 125 (CA 125) and carcinoembryonic antigen (CEA) are used in routine practice to help CCA diagnosis and mostly to monitor disease progression once diagnosis has been achieved [13]. The major limitation is the low sensitivity and specificity of these biomarkers, thus not allowing the early detection of CCA [12]. In a subgroup of patients with PSC, a CA 19-9 value exceeding 129 U/mL was found to have a specificity and sensitivity of 98 and 79% for CCA diagnosis, respectively [24]. However, CA 19-9 is not specific of CCA and elevations can be also linked to other benign or malignant pathologies [25]. Furthermore, CA 19-9 is not detectable in 7% of the general population due to the absence of the blood cell Lewis antigen [26]. Similarly, other serum biomarkers (e.g., cytokeratin-19 fragment and CA-242) have been reported to be superior than CA 19-9 for CCA but are not yet available in clinical practice [12].

Another important field of biomarkers research relies on the differential diagnosis between HCC and iCCA. CA 19-9, sialic acid, and CA 242 serum levels tend to be increased in patients with CCA, while α-fetoprotein and glypican-3 are most exclusively increased in HCC [12]. However, the differential diagnosis of both primary liver tumors, particularly when facing tumors with combined features, such as mixed HCC-iCCA tumors, is difficult.

### 2.2. CCA Treatment and Prognosis

The potentially curative treatment of choice for CCA is surgical resection. There are few serum biomarkers used in clinical practice to predict evolution after surgery. High CA 19-9 or gamma glutamyl transpeptidase levels are usually associated with bigger tumors, lymph node metastasis or vascular invasion. However, no cut-off value has been validated to contraindicate surgery in patients with CCA [12]. Still, increased levels of CA19-9 (>1000 U/mL) have been related with metastatic CCA, making this biomarker potentially useful to alert the possibility of advanced disease stage [16]. In pCCA tumors, there are no validated biomarkers used in CCA staging or surgery evaluation. Five-year survival rates after surgical resection with negative margins range from 11% to 41% [27]. Neoadjuvant chemoradiation before liver transplantation is an option in very selected patients with pCCA, [1,25] as set out by Murad et al. [1,25]. Finally, distinguishing dCCA from early pancreatic cancer can be challenging due the anatomical proximity, histopathological similarity and the lack of biomarkers that can effectively differentiate both tumors.

Until the recent (2020) approval of pemigatinib as the first targeted therapy for advanced iCCA with *FGFR2* fusions, the use of biomarkers to guide therapy was less studied previously [12]. However, several therapeutic agents that are currently in clinical trials have specific targets that are expressed in CCA tumors, implying a great opportunity for research on prognostic, predictive and pharmacodynamic biomarkers.

## 3. Non-Invasive Biomarkers

### 3.1. Circulating Nucleic Acids

#### 3.1.1. Circulating Tumor DNA

Tumor biopsies are still the gold standard for cancer diagnosis and the primary method for molecular testing for genetic alterations. In CCA however, it can be challenging to obtain enough tissue for comprehensive molecular testing or even diagnosis, especially in patients with pCCA. The evaluation of solid tumor malignancies through the analysis of cell free DNA (cfDNA), which can be efficiently isolated and analyzed using advanced methods such as digital droplet PCR or next-generation sequencing is now envisioned. Circulating tumor DNA (ctDNA) is released into the circulation from apoptotic and/or necrotic tumor cells and is usually detected as 150–200 base pairs, double-stranded fragments. ctDNA has been shown to carry tumor-specific (epi)genetic alterations and is therefore particularly attractive to assess the patient-specific tumoral mutational background in a non-invasive and accurate manner. Detecting ctDNA might be helpful for: (1) early detection of disease; (2) monitoring of patients at risk for cancer development; (3) identification of therapeutic targets and guide therapeutic decisions (personalized medicine); (4) evaluation of treatment response, including prediction of prognosis (tumor relapse and disease progression); and (5) help to understand primary and secondary mechanisms of drug resistance. The portion of ctDNA in cfDNA in patients with cancer varies greatly from less than 1% to more than 90%. In the context of biliary tract cancers (BTCs), ctDNA was easily detected and allowed the identification of genomic alterations in 89% of patients, thus pinpointing the relevance of ctDNA in these types of cancers [28].

Advances in genomic profiling techniques are continuously helping in the identification of genetic alterations that drive carcinogenesis across multiple tumor types including CCA, which include activating point mutations, fusions or rearrangements, amplifications, and/or deletions. Overall, multiple studies have revealed that CCAs are highly heterogeneous at the genetic level, and specific molecular profiles are observed according to the anatomical location and the histological subtype. Despite the genetic diversity, a recurrent repertoire of mutations in driver genes and potentially targetable aberrations are evident. Indeed, several studies suggested that approximately 40% of patients display targetable genetic alterations (e.g., *KRAS*, *TP53*, *IDH1* and *IDH2*, *CDKN2A/B* and less commonly in *ERBB2/3*, *MET*, *BRAF* and *PIK3CA*) [29,30,31,32,33]. Of note, the first precision oncology-based randomized phase III study in patients with iCCA harboring *IDH1* mutations treated with the IDH-inhibitor ivosidenib reported promising data, meeting the primary endpoint (improved progression-free survival) and highlighting the importance of accurate genetic testing in CCA [34,35]. Furthermore, *fibroblast growth factor receptor 2* (*FGFR2*) fusions are found in 10–15% of patients with iCCA and results from the phase II FIGHT-202 study led to the recent Food and Drug Administration (FDA) and European Medicines Agency (EMA) approval of the FGFR inhibitor pemigatinib for the treatment of iCCA based on high radiological response rates in pre-treated patients with durability of the observed responses [36]. Several recent studies have confirmed the feasibility of ctDNA testing to detect these genetic alterations in CCA and GBC with a high concordance rate to tissue biopsies [37,38,39,40]. In addition, it has been recently shown that ctDNA may not only be used to identify patients with *FGFR2* fusions, but can also aid the tracking of polyclonal clones that drive acquired FGFR inhibitors resistance during therapy [41]. Real-time detection of clonal evolution of mutations directly linked with therapy resistance may therefore be a valuable tool to guide sequential therapy since distinct FGFR inhibitors have varying abilities to overcome this secondary resistance. Of note, it is not only blood that can be used for a liquid-biopsy approach in CCA; bile also represents a promising source of ctDNA that deserves attention in the future [42].

#### 3.1.2. Cell-Free Non-Coding RNAs

Cell-free non-coding RNAs have been under the spotlight as promising non-invasive biomarkers for several diseases, including cancer and CCA. In this sense, microRNAs (miRNAs or miRs) have gained significant attention in this field since they are highly abundant and stable in biofluids, being more resistant to degradation and/or modification and being easily detected and amplified [43]. In order to understand the diagnostic value of miRNAs for CCA, two meta-analyses have been conducted, reporting pooled area under of the receiver operator curve (AUC) values of ~0.9 [44,45]. In these studies, bile miRNAs were reported with the highest diagnostic capacity (AUC of 0.95), followed by serum (0.913) and urine (0.745) [44].

Several miRNAs have already been found in abundance in several biological fluids, embodying a diagnostic and/or prognostic value for CCA (Table 1). In bile, miR-9 and miR-145 were found to be increased in the serum of patients with CCA compared to controls (patients with choledocholithiasis), with an AUC value of 0.975 [46]. Additionally, the levels of miR-412, -640, -1537 and -3189 were reported to be increased in the bile of patients who developed CCA on a PSC background, when compared with patients with isolated PSC, allowing their differential diagnosis with good diagnostic values (~0.8 for all). Notably, combining miR-1537 with serum CA19-9 levels improved the diagnostic capacity of CA19-9 alone for the diagnosis of CCA [47]. More recently, levels of bile-derived circulating miR-30d-5p and miR-92a-3p were found upregulated in patients with CCA, when compared with patients with benign biliary disease (AUC of 0.730, and 0.652, respectively), although superiority over CA19-9 and CEA was not confirmed [48]. In serum (and plasma), the levels of the well-known oncomiR-21 were consistently found upregulated in patients with CCA, compared to healthy controls, displaying high diagnostic values (AUC ~0.9) and also correlating positively with advanced tumor stage (TNM) and worse overall survival [49,50]. Additionally, increased serum miR-21 and miR-221 levels were reported in patients with hepatolithiasis-associated CCA, compared to patients with isolated hepatolithiasis, but their diagnostic value was limited (AUC: 0.610 and 0.767, respectively). Nevertheless, combining both of them with ultrasound-related findings (liver abscess, echotexture, border demarcation and portal vein around lesion) significantly ameliorated their diagnostic capacity (AUC 0.911), thus allowing the specific diagnosis of CCA in patients with hepatolithiasis [51]. The levels of urinary miR-21 were also found increased in patients with *O. viverrini*-related CCA, when compared with healthy controls, and the combination with the augmented levels of miR-192 improved the diagnostic capacity of each one of them than when evaluated alone, harboring an AUC value of 0.849 [52]. Additionally, a prognostic value for serum miR-21 was also proposed since its levels reduced after tumor resection [53], thus potentially positioning miR-21 both as a diagnostic and prognostic biomarker. Still, the translation of these findings into clinics should be conducted with caution since miR-21 has also been reported to be upregulated in the serum of patients with HCC [54,55,56,57] and other types of cancer [58,59,60,61], constituting probably a pan-cancer diagnostic biomarker.

Decreased levels of serum miR-106a appear to aid CCA diagnosis with great accuracy (AUC of 0.890), also predicting poor prognosis and a higher likelihood of lymph node metastasis [62]. Furthermore, increased serum levels miR-483-5p might also help with the diagnosis of CCA when compared either with healthy controls (AUC: 0.770) or patients with PSC (AUC of 0.700) but its diagnostic capacity improves when combined with miR-194 (combined AUC of 0.810) or with miR-222 (combined AUC of 0.770), for the discrimination of controls or PSC, respectively [63].

Levels of serum miR-122, -192, -29 and -155 were shown more abundantly present in patients with CCA, compared to either healthy controls (AUC of at least 0.664) or patients with PSC (AUC of at least 0.787). Although none of these miRNAs were superior than CA19-9 for the differential diagnosis of CCA and PSC, serum miR-122 display a higher diagnostic capacity when compared to this tumor biomarker, providing an AUC value of 0.992 when comparing patients with CCA and healthy controls. Interestingly, miR-192 serum post-surgery levels predicted a worse prognosis in these patients as the levels of serum miR-122 declined after tumor resection, and correlated with a better median cumulative survival [64]. The diagnostic and prognostic value of serum miR-192 was further confirmed in another study in which increased levels were reported in patients with *O. viverrini*-related CCA compared with healthy controls, presenting high accuracy (AUC of 0.803) and positively correlating with lymph node metastasis and worse survival. This study also confirmed the diagnostic value of serum miR-21 and further reported increased levels of miR-150 in patients with CCA, while miR-26a was found reduced. In fact, contradictory findings regarding these two miRNAs have been published. Whereas one study confirmed the upregulation of miR-150 in patients with iCCA, compared with individuals without cancer (AUC of 0.764, being further improved when combined with CA19-9) [65], two other studies including patients with CCA, PSC and healthy controls revealed decreased serum levels of miR-150, although no diagnostic values were provided [66,67]. Similarly, levels of miR-26a were found to be increased in patients with CCA in comparison to healthy controls, displaying an AUC value of 0.899, further positively correlating with clinical stage, tumor differentiation status and metastasis and also with worse prognosis (poor survival) [68] although diminished levels were detected in the serum of other patients with CCA, compared with PSC (AUC of 0.780) [47]. In these previous studies, the authors also identified other decreased serum miRNAs, namely miR-1281 (AUC of 0.830), miR-126 (AUC of 0.870), miR-30b (AUC of 0.780) and particularly miR-122 (AUC of 0.650), which also contrasts with previous reports published for this last miRNA. Consequently, questions arise regarding their usefulness as diagnostic and/or prognostic biomarkers for all forms of the disease and specific risk factors and validation studies are warranted in the future with well-defined and large international cohorts of patients.

Overall, several individual miRNAs were shown to provide high diagnostic capacity for CCA but validation studies with highly selected and larger cohorts of patients (including proper control groups) are now necessary. Specifically, the validation of circulating levels (serum and urine) of miR-21 and miR-192 would be of great interest since their diagnostic capacity have been highlighted in several studies with large cohorts of patients (Table 1). Furthermore, increasing evidence points towards the usefulness of specific panels of miRNAs for the diagnosis of CCA, due to their greater sensitivity and specificity when compared to individual miRNAs. In this sense, a plasma miRNA profile comprised of 8 miRNAs (miR-483-5p, 505-3p, -874, -885-5p, -320b, -92b-3p, -1275 and -1307-3p), was associated with the presence of *O. viverrini*-related iCCA [69]. Still, the diagnostic parameters of this panel were not calculated. Nevertheless, development of novel miRNA-related diagnostic and/or prognostic panels are warranted in the near future.

**Table 1:** Circulating miRNAs with potential diagnostic value for CCA.

Source	miRNA	Levels	Comparison	AUC	Ref.
Serum/plasma	miR-21	Up	iCCA (*n* = 74) vs. healthy controls (*n* = 74)	0.908	[49]
		Up	iCCA (*n* = 25) vs. healthy controls (*n* = 7)	0.940	[50]
		Up	Hepatolithiasis-CCA (*n* = 31) vs. hepatolithiasis (*n* = 40)	0.610	[51]
	miR-221	Up	Hepatolithiasis-CCA (*n* = 31) vs. hepatolithiasis (*n* = 40)	0.767	[51]
	miR-106a	Down	CCA (*n* = 103) vs. healthy controls (*n* = 20)	0.890	[62]
	miR-194 and miR-483-5p	Up	CCA (*n* = 30) vs. healthy controls (*n* = 30)	0.810	[63]
	miR-222 and miR-483-5p	Up	CCA (*n* = 30) vs. PSC (*n* = 30)	0.770	
	miR-122	Up	CCA (*n* = 94) vs. healthy controls (*n* = 40)	0.992	[64]
		Down	CCA (*n* = 30) vs. PSC (*n* = 30)	0.650	[47]
	miR-192	Up	*O. viverrini*-related CCA (*n* = 51) vs. healthy controls (*n* = 32)	0.803	[70]
	miR-150	Up	iCCA (*n* = 15) vs. healthy controls (*n* = 15)	0.764	[65]
	miR-26a	Up	CCA (*n* = 66) vs. healthy controls (*n* = 66)	0.899	[68]
		Down	CCA (*n* = 30) vs. PSC (*n* = 30)	0.780	[47]
	miR-1281	Down	CCA (*n* = 31) vs. PSC (*n* = 40)	0.830	[47]
	miR-126	Down	CCA (*n* = 30) vs. PSC (*n* = 30)	0.870	
	miR-30b	Down	CCA (*n* = 30) vs. PSC (*n* = 30)	0.780	
Bile	miR-9	Up	Biliary tract cancer (*n* = 9) vs. choledocholithiasis (*n* = 9)	0.975	[46]
	miR-145	Up	Biliary tract cancer (*n* = 9) vs. choledocholithiasis (*n* = 9)	0.975	[46]
	miR-640	Up	PSC-CCA (*n* = 12) vs. PSC (*n* = 52)	0.810	[47]
	miR-412	Up	PSC-CCA (*n* = 12) vs. PSC (*n* = 52)	0.810	[47]
	miR-1537	Up	PSC-CCA (*n* = 12) vs. PSC (*n* = 52)	0.780	[47]
	miR-3189	Up	PSC-CCA (*n* = 12) vs. PSC (*n* = 52)	0.800	[47]
	miR-30d-5p	Up	CCA (*n* = 37) vs. obstructive benign biliary disease (*n* = 48)	0.730	[48]
	miR-92a-3p	Up	CCA (*n* = 37) vs. obstructive benign biliary disease (*n* = 48)	0.652	[48]
Urine	miR-21 and miR-192	Up	CCA (*n* = 22) vs. healthy controls (*n* = 21)	0.849	[52]

### 3.2. Cytokines/Proteins

CA19-9 and carcinoembryonic antigen (CEA) constitute the most widely used protein biomarkers for diagnosis and monitoring of CCA. Still, as aforementioned, the sensitivity and specificity values are far from satisfactory [71,72,73,74]. Novel promising circulating diagnostic and prognostic protein biomarkers are displayed in Table 2. Cytokeratin-19 fragment (CYFRA 21-1) was previously reported to be elevated in the serum of patients with iCCA when compared with individuals with benign biliary diseases, with sensitivity and specificity values of 75.6% and 96.2%, respectively. CYFRA 21-1 presented a higher diagnostic capacity compared with either CA19-9 or CEA and positively correlated with tumor stage, representing an independent predictor of reduced recurrence-free and overall survival [75,76]. Similarly, serum matrix metalloproteinase-7 (MMP-7) levels also allowed the differential diagnosis of CCA and patients with benign biliary diseases with high accuracy (AUCs of 0.730 and 0.840) [77,78] but its relation with prognosis remains to be unveiled. The circulating levels of glycol phosphoprotein osteopontin were found markedly increased in patients with CCA, compared with either healthy controls (AUC of 0.964) and patients with PSC, providing a higher diagnostic accuracy than CA19-9 or CEA. High pre- and post-surgery levels of osteopontin were also positively associated with reduced overall survival after tumor resection, thus proving also a prognostic value [79]. More recently, the N-glycoproteome of plasma obtained from patients with CCA was studied and the levels of galectin-3 binding protein were highly correlated with tumor stage and grade, recurrence-free and overall survival but the diagnostic value of this circulating protein remains to be elucidated [80]. On the other hand, a clear diagnostic and prognostic value for soluble urokinase plasminogen activator receptor (suPAR) was recently reported [81]. The authors found that the serum levels of suPAR were markedly increased in patients with biliary tract cancer, when compared with healthy controls (AUC of 1.000 and 0.969 in discovery and validation cohorts, respectively) and also with patients with PSC (AUC of 0.719). Moreover, an optimal prognostic value of 3.72 ng/mL for patients with biliary tract cancer was defined with a high suPAR shown to be an independent prognostic predictor of tumor stage and worse prognosis (overall survival and acute kidney injury after tumor resection). Additionally, the levels of other protein biomarkers, including S100 calcium binding protein A6 (S100A6) [82,83], dickkopf-related protein 1 (DKK1) [84] and SSP411 [85] were found increased in patients with CCA, but their diagnostic value remains to be clarified.

The levels of circulating cytokines might also contribute for the diagnosis of CCA. Increased serum levels of the pro-inflammatory cytokine interleukin-6 (IL-6), usually secreted by CCA cells [86], were reported in patients with bile duct cancer compared to healthy individuals, harboring great sensitivity (73%) and specificity (92%) values and also being proposed as a potential marker for therapy monitoring [87]. More recently, the levels of transforming growth factor β 1 (TGF-β1) in the serum of patients with CCA were reported elevated, when compared with healthy controls, although the diagnostic capacity was not remarkable (AUC of 0.668). However, increased TGF-β1 levels were correlated with the presence of metastasis, arising as a new potential metastatic biomarker for CCA (cut-off value: 48.95 ng/mL; sensitivity: 48.2% and specificity: 88.9%) [88].

**Table 2:** Circulating protein/cytokines with potential diagnostic value for CCA.

Source	Protein/Ctyokine	Levels	Comparison	AUC	Ref.
Serum/plasma	CYFRA 21-1	Up	Biliary tract cancer (*n* = 134) vs. benign biliary diseases (*n* = 52)	0.851	[75]
	MMP7	Up	CCA (*n* = 44) vs. benign biliary tract disease (*n* = 36)	0.730	[78]
		Up	CCA (*n* = 59) vs. benign biliary tract disease (*n* = 128)	0.840	[77]
	Osteopontin	Up	CCA (*n* = 80) vs. healthy controls (*n* = 42)	0.964	[79]
	IL-6	Up	Bile duct cancer (*n* = 26) vs. healthy controls (*n* = 23)	0.875	[87]
	S100A6	Up	CCA (*n* = 29) vs. healthy controls (*n* = 22)	0.909	[82]
	DKK1	Up	iCCA (*n* = 37) vs. healthy controls (*n* = 50)	0.872	[84]
	SSP411	Up	CCA (*n* = 35) vs. “cholangitis (*n* = 13) and healthy controls (*n* = 23)”	0.913	[85]
	suPAR	Up	Biliary tract cancer (*n* = 95) vs. healthy controls (*n* = 66)	0.969	[81]
	TGF-β1	Up	CCA (*n* = 45) vs. healthy controls (*n* = 45)	0.668	[88]

### 3.3. Metabolites

Tumor development and growth are associated with a myriad of metabolic changes including dysregulated lipid and amino acid metabolism as well as perturbation of glycolysis that determine variations in the content of multiple metabolites in biofluids (i.e., serum, bile or urine) that can be detected with highly sensitive techniques (“metabolomics”) [89]. In the case of liver cancer, chronic cholestasis and inflammation, leading to oxidative stress and lipid peroxidation, contribute to these changes along with genetic and epigenetic changes occurring during carcinogenesis [1]. Indeed, identification of tumor-related metabolite signatures in biological samples obtained from patients at risk (i.e., PSC patients) holds promise for development of robust biomarkers of CCA [90]. Data on metabolomics in CCA is rapidly emerging and this opens the possibility to define signatures allowing early diagnosis (Table 3). In addition, metabolomic data could also be used in combination with other determinations (i.e., multi-omics approaches) to improve their diagnostic performance [90]. Once validated, these tests may also be of help in the assessment of prognosis or treatment monitoring. In addition to tumor detection, differentiation between iCCA and p/dCCA may also be possible through serum metabolomics.

With regard to lipids, in a study from China [91], serum levels of LysoPC (14:0, C_22_H_46_NO_7_P) and LysoPC (15:0, C_23_H_48_NO_7_P) were found to be reduced while levels of 21-deoxycortisol and bilirubin were significantly increased in the serum of patients with CCA compared with controls, suggesting that combination of these four metabolites could aid CCA diagnosis. In another study, serum metabolomic profiling of patients with biopsy-proven iCCA, HCC and PSC and from healthy individuals was conducted [92]. Data allowed the design of an algorithm that differentiated iCCA from HCC with an AUC: 0.9 and good sensitivity (80%) and specificity (90%) values. This algorithm combined three sphingomyelins (SMs), two phosphatidylcholines (PCs) and one ceramide (Cer). Moreover, a second algorithm was designed, combining PC (34:3) and histidine, that differentiate PSC from CCA with an excellent accuracy value [92]. Of note, these results were later validated in an independent cohort of patients. Additionally, a recent comprehensive analysis of lipids, bile acids and small molecules was carried out in bile from patients with CCA and compared to patients with benign strictures and patients with biliary obstruction due to pancreatic cancer and further artificial intelligence-based technology was utilized to select biomarkers [93]. This allowed the combination of lipid species able to differentially diagnose patients with benign stenoses and CCA with high sensitivity and specificity (94.1% and 92.3%, respectively; AUC of 0.984). Among the species identified were several phosphatidylcholines, certain ceramides and total TG levels. These findings need to be validated in larger groups of patients. Finally, in a recent study from the UK, no significant differences were found between serum phospholipid profiles from patients with CCA and benign biliary strictures [94].

The search for CCA metabolite biomarkers in urine has been also explored. Of note, one study found that four metabolites (creatine riboside, N-acetylneuraminic acid, cortisol sulfate and a lipid molecule) were elevated in patients with CCA and HCC, using serum CA19-9 levels to differentiate between them (AUC: 0.88) [95].

### 3.4. Extracellular Vesicles

In the last decade, extracellular vesicles (EVs) opened a new field in the quest to find non-invasive biomarkers for hepatobiliary malignancies [96]. EVs comprise a group of small membrane-encapsulated spheres that are released from every cell type and potentially detected in all biological fluids (blood, saliva, urine and bile) [97,98,99,100]. According to their biogenesis and size, EVs might be classified into exosomes, microvesicles/microparticles and apoptotic bodies [101,102]. They contain a variety of biomolecules, such as proteins, nucleic acids lipids and metabolites through which they mediate cell–cell communication and constitute a rich source of novel specific non-invasive biomarkers. In CCA, increased bile and serum EV concentration was firstly described to allow the differential diagnosis between malignant (pancreatic cancer and CCA) and non-malignant common bile duct stenosis (chronic pancreatitis) with the highest diagnostic capacity for bile (AUC of 1.000) and with sensitivity values of 47% for serum [103]. Additionally, the presence of AnnexinV/EpCAM/ASGPR1^+^ tumor-associated microparticles (TAMPs) in the serum of patients with CCA or liver cancer (CCA and HCC) allowed their diagnosis, when compared with patients with liver cirrhosis (AUC of 0.630 and 0.700, respectively) although it did not differentiate between the two main types of primary liver cancer [104]. Of note, the levels of TAMPs markedly reduced one week after tumor resection, showing an important relation of these EVs and the presence of the tumor.

**Table 3:** Circulating metabolites with potential diagnostic value for CCA.

Source	Metabolite	Levels	Comparison	AUC	Ref.
Serum	21-Deoxycortisol	Down	CCA (*n* = 225) vs. healthy controls (*n* = 101)	0.918	[91]
	Bilirubin	Up	CCA (*n* = 225) vs. healthy controls (*n* = 101)	0.922	[91]
	LysoPC (14:0)	Down	CCA (*n* = 225) vs. healthy controls (*n* = 101)	0.954	[91]
	LysoPC (15:0)	Up	CCA (*n* = 225) vs. healthy controls (*n* = 101)	0.927	[91]
	Glycocholic acid	Up	Biopsy-proven iCCA (*n* = 20) vs. healthy controls (*n* = 20)	DIS: 0.857 VAL: 0.991	[92]
	Glycochenodeoxycholic acid	Up	Biopsy-proven iCCA (*n* = 20) vs. healthy controls (*n* = 20)	DIS: 0.823 VAL: 0.987	[92]
	Androsterone sulfate II	Down	Biopsy-proven iCCA (*n* = 20) vs. healthy controls (*n* = 20)	DIS: 0.808 VAL: 0.800	[92]
	Dehydroepiandrosterone	Up	Biopsy-proven iCCA (*n* = 20) vs. healthy controls (*n* = 20)	DIS: 0.790 VAL: 0.804	[92]
	ChoE (22:6)	Down	Biopsy-proven iCCA (*n* = 20) vs. healthy controls (*n* = 20)	DIS: 0.763 VAL: 0.769	[92]
	ChoE (20:4)	Down	Biopsy-proven iCCA (*n* = 20) vs. healthy controls (*n* = 20)	DIS: 0.760 VAL: 0.778	[92]
	CMH (d18:1/16:0)	Up	Biopsy-proven iCCA (*n* = 20) vs. healthy controls (*n* = 20)	DIS: 0.798 VAL: 0.809	[92]
	PC (16:0/16:0)	Up	Biopsy-proven iCCA (*n* = 20) vs. healthy controls (*n* = 20)	DIS: 0.773 VAL: 0.920	[92]
	SM(43:2) PC(O-16:0/20:3) PC(O-18:0/18:2) SM(d18:2/16:0) Cer(d18:1/16:0) SM(42:3)	Up Down Down Up Up Up	iCCA (*n* = 20) vs. HCC (*n* = 20) (Biopsy-proven patients)	DIS:0.900 VAL: 0.981	[92]
	PC(34:3) Histidine	Down	iCCA (*n* = 20) vs. PSC (*n* = 20) (Biopsy-proven patients)	DIS: 0.990 VAL: 0.995	[92]
Bile	Phosphatidylcholine Bile acids Cholesterol/lipid	Down	CCA (*n* = 16) vs. begin non-PSC biliary diseases (*n* = 27)	SEN: 88.9% SPE: 87.1%	[105]
	Glycine-conjugated bile acids Phosphatidylcholines	Up Down	“Inoperable pCCA (*n* = 3) and dCCA (*n* = 2)” vs. non-malignant biliary diseases without cholestasis (*n* = 20)	SEN: 80% SPE: 95%	[106]

Proteomic analysis of EVs isolated from serum of patients with liver cancer (HCC or CCA), PSC and healthy individuals provided new potential protein non-invasive biomarkers with high diagnostic capacity (Table 4) [107]. For instance, polymeric immunoglobulin receptor (PIGR), aminopeptidase N (AMPN) and pantetheinase (VNN1) allowed the early and accurate diagnosis of CCA, when compared with healthy controls (AUC of 0.905, 0.833 and 0.833, respectively). Furthermore, ficolin-2 (FCN2), inter-α-trypsin inhibitor heavy chain H4 (ITIH4) and fibrinogen γ chain (FIBG) provided high diagnostic values for the differential diagnosis of patients with early stage CCA and PSC, being superior diagnostic biomarkers than CA19-9. Moreover, several EV proteins, including FIBG, α-1-acid glycoprotein (A1AG1) and vitamin D binding protein (VTDB) enabled the differential diagnosis of iCCA and HCC, with greater diagnostic power when compared with either CA19-9 or AFP. More recently, transcriptomic (messenger RNA (mRNA) and non-coding RNAs) studies in serum and urine EVs isolated from patients with CCA, PSC, ulcerative colitis (UC) and healthy individuals provided new transcript liquid biopsy biomarkers with accurate values [108]. In fact, when comparing the specific mRNA profiles of serum and urine EVs obtained from patients with CCA with the transcriptome data from two independent international cohorts of patients (The Cancer Genome Atlas and the Copenhagen cohorts) and from CCA cells in vitro, as well as from CCA cells-derived EVs, 105 and 39 commonly altered transcripts were identified, respectively. Interestingly, gene ontology analysis revealed that all these transcripts might be involved in key cellular processes during cholangiocarcinogenesis. In serum, the most promising liquid biopsy biomarkers were c-Maf inducing protein (*CMIP*), glutamate decarboxylase 1 (*GAD1*), nucleoside diphosphate kinase 1 (*NME1*), CDP-diacylglycerol synthase 1 (*CDS1*), and cyclin-dependent kinases regulatory subunit 1 (*CKS1B*), showing AUC values of 0.957, 0.928, 0.899, 0.893, and 0.891, respectively, for the diagnosis of CCA in comparison with control group while the combination of *CMIP*, *NME1* and *CKS1B* provided the maximum diagnostic capacity (AUC: 1.000). On the other hand, in urine, the transcripts ubiquitin conjugating enzyme E2 C (*UBE2C*) and serine protease inhibitor B1 (*SERPINB1*) arose as novel potential liquid biopsy biomarkers and the combination of these two transcripts provided an AUC value of 0.812 for the diagnosis of CCA. This type of analysis allowed, for the first time, the identification of new non-invasive RNA biomarkers with high diagnostic capacity that also mirror their levels in the tumor tissue, thus constituting a novel and innovative liquid biopsy approach. Importantly, some of these liquid biopsy biomarkers correlated with clinicopathological findings in patients with CCA, and the relevance of them in predicting disease prognosis and/or helping in the decision of therapeutic regimens is eagerly awaited [108].

EVs might also be a source of non-coding RNAs that are amenable to be detected and that might harbor diagnostic and/or prognostic value. In this sense, the analysis of the transcriptome of bile EVs revealed the increased levels of two long non-coding RNAs (ENST00000588480.1 and ENST00000517758) in patients with CCA, compared with healthy controls [109]. Combining the levels of these two long non-coding RNAs provided an AUC value of 0.709, positively correlating with disease stage and worse overall survival. Similarly, the levels of other non-coding RNAs, including miRNAs, long non-coding RNAs, small nuclear RNAs, among others, were detected in serum and urine EVs and described to harbor great diagnostic values (AUC up to 0.909 in serum and 0.830 in urine) [108]. Additionally, a panel containing 5 different miRNAs (miR-191, -486-3p, -1274b, -16 and -484) was augmented in bile EVs from patients with CCA, when compared with patients with PSC, biliary obstruction and biliary leak, with sensitivity and specificity values of 67% and 96%, respectively [110]. More recently, serum EVs miR-200 family (miR-141-3p, -200a-3p, 200b-3p and 200c-3p) were reported markedly increased in patients with CCA, when compared with healthy controls, providing a higher diagnostic value than CA19-9 [111]. Particularly, serum EVs miR-200c-3p presented the highest AUC value (0.93) and positively correlated with tumor stage, being more elevated in patients with stage III-IV, in comparison to stage I-II. Similarly, increased levels of miR-96-5p, -151a-5p, -191-5p and 4732-3p in serum EVs were reported to have a diagnostic value for CCA (AUC of 0.733, 0.764, 0.542 and 0.654, respectively) [112] although it has yet to be shown if they have superior diagnostic value than CA19-9 for the identification of bile duct cancer.

**Table 4:** Circulating extracellular vesicles containing biomolecules with potential diagnostic value for CCA.

Source	EV Cargo	Biomarker Type	Levels	Comparison	AUC	Ref.
Serum	AnnexinV^+^ EpCAM^+^ ASGPR1^+^	TAMP concentration	Up	CCA (*n* = 38) vs. cirrhosis (*n* = 49)	0.630	[104]
	AMPN	Protein	Up	CCA (*n* = 43) vs. healthy controls (*n* = 32)	0.878	[107]
	VNN1		Up	CCA (*n* = 43) vs. healthy controls (*n* = 32)	0.876	
	PIGR		Up	CCA (*n* = 43) vs. healthy controls (*n* = 32)	0.844	
	PIGR		Up	Early stage CCA (*n* = 13) vs. healthy controls (*n* = 22)	0.905	
	AMPN		Up	Early stage CCA (*n* = 13) vs. healthy controls (*n* = 22)	0.833	
	FIBG		Up	Early stage CCA (*n* = 13) vs. healthy controls (*n* = 22)	0.833	
	FIBG		Up	CCA (*n* = 43) vs. PSC (*n* = 30)	0.796	
	A1AG1		Up	CCA (*n* = 43) vs. PSC (*n* = 30)	0.794	
	S100A8		Up	CCA (*n* = 43) vs. PSC (*n* = 30)	0.759	
	FCN2		Up	Early stage CCA (*n* = 13) vs. PSC (*n* = 30)	0.956	
	ITIH4		Up	Early stage CCA (*n* = 13) vs. PSC (*n* = 30)	0.881	
	FIBG		Up	Early stage CCA (*n* = 13) vs. PSC (*n* = 30)	0.881	
	FIBG		Up	iCCA (*n* = 12) vs. HCC (*n* = 29)	0.894	
	A1AG1		Up	iCCA (*n* = 12) vs. HCC (*n* = 29)	0.845	
	VTDB		Up	iCCA (*n* = 12) vs. HCC (*n* = 29)	0.823	
	*CMIP*	RNA	Up	CCA (*n* = 12) vs. (PSC + UC + healthy controls) (*n* = 23)	0.957	[108]
	*GAD1*		Up	CCA (*n* = 12) vs. (PSC + UC + healthy controls) (*n* = 23)	0.928	
	*NME1*		Up	CCA (*n* = 12) vs. (PSC + UC + healthy controls) (*n* = 23)	0.899	
	*CDS1*		Up	CCA (*n* = 12) vs. (PSC + UC + healthy controls) (*n* = 23)	0.893	
	*CKS1B*		Up	CCA (*n* = 12) vs. (PSC + UC + healthy controls) (*n* = 23)	0.891	
	*CMIP* *NME1* *CKS1B*		Up	CCA (*n* = 12) vs. (PSC + UC + healthy controls) (*n* = 23)	1.000	
	miR-551B	miRNA	Up	CCA (*n* = 12) vs. (PSC + UC + healthy controls) (*n* = 23)	0.909	[108]
	*PMS2L4*	pseudogene	Up	CCA (*n* = 12) vs. (PSC + UC + healthy controls) (*n* = 23)	0.880	[108]
	*LOC643955*	pseudogene	Up	CCA (*n* = 12) vs. (PSC + UC + healthy controls) (*n* = 23)	0.873	[108]
	*LOC100134868*	lncRNA	Up	CCA (*n* = 12) vs. (PSC + UC + healthy controls) (*n* = 23)	0.864	[108]
	*PTTG3P*	pseudogene	Up	CCA (*n* = 12) vs. (PSC + UC + healthy controls) (*n* = 23)	0.859	[108]
	miR-200c-3p	miRNA	Up	CCA (*n* = 36) vs. healthy controls (*n* = 12)	0.930	[111]
	miR-96-5p		Up	CCA (*n* = 45) vs. healthy controls (*n* = 40)	0.733	[112]
	miR-151a-5p		Up	CCA (*n* = 45) vs. healthy controls (*n* = 40)	0.764	
	miR-191-5p		Up	CCA (*n* = 45) vs. healthy controls (*n* = 40)	0.542	
	miR-4732-3p		Up	CCA (*n* = 45) vs. healthy controls (*n* = 40)	0.654	
Urine	*UBE2C*	RNA	Up	CCA (*n* = 23) vs. (PSC + UC + healthy controls) (*n* = 22)	0.779	[108]
	*SERPINB1*		Up	CCA (*n* = 23) vs. (PSC + UC + healthy controls) (*n* = 22)	0.654	
	*UBE2C* *SERPINB1*		Up	CCA (*n* = 23) vs. (PSC + UC + healthy controls) (*n* = 22)	0.812	
	*RNU11*	snRNA	Up	CCA (*n* = 23) vs. (PSC + UC + healthy controls) (*n* = 22)	0.830	[108]
	*LOC257358*	miscRNA	Up	CCA (*n* = 23) vs. (PSC + UC + healthy controls) (*n* = 22)	0.812	[108]
	*VTRNA1-1*	vtRNA	Up	CCA (*n* = 23) vs. (PSC + UC + healthy controls) (*n* = 22)	0.777	[108]
	*AURKAPS1*	Pseudogene	Down	CCA (*n* = 23) vs. (PSC + UC + healthy controls) (*n* = 22)	0.771	[108]
	miR-483	miRNA	Down	CCA (*n* = 23) vs. (PSC + UC + healthy controls) (*n* = 22)	0.763	[108]
Bile	miR-191 miR-486-3p miR-1274b miR-16 miR-484	miRNA	Up	CCA (*n* = 46) vs. benign biliary diseases (*n* = 50)	SEN: 67% SPE: 96%	[110]

### 3.5. Circulating Tumor Cells

Intact tumor cells intravasate into the bloodstream at low frequency (often <10 circulating tumor cells (CTCs) per mL of blood in patients with metastatic cancer). The abundance of these CTCs in liquid biopsies from patients with cancer varies between different tumor types, and so far, no tumor specific biomarker has been identified that unequivocally distinguishes CTCs from normal cells.

Multiple techniques have been developed to isolate CTC from blood, including enrichment based on morphological markers (e.g., size and shape of the cells), as well as positive enrichment using surface markers such as epithelial cell adhesion molecule (EpCAM) [113]. For detection of CCA-derived CTCs, EpCAM appears to be a valid marker since the majority of CCAs express EpCAM [114] although only 10–20% of these patients display an upregulation of EpCAM levels, when compared with non-tumoral tissue [115], therefore limiting the applicability of this platform for CCA. Consequently, the number of cells identified by this approach may underestimate the true prevalence of CTCs. A recent study reported a significantly higher prevalence of CTCs in patients with biliary tract cancer by applying a protocol that allows the identification not only of epithelial CTCs but also of “nonconventional” CTCs which lack epithelial and leukocyte markers but which display genomic alterations [116]. Although the prognostic impact of these different CTC subpopulations needs to be validated in confirmatory studies, they may become a useful tool for clinical decision making in CCA.

The prognostic value of CTCs has been addressed/confirmed in a variety of malignancies, including breast, prostate and colon cancer. On the other hand, only a few studies investigated the correlation between the presence of CTCs and survival of patients with CCA. In one of the largest studies, CTCs were detected in 26% of BTC (>2 per 7.5 mL of blood), serving as an independent predictor of survival [117]. The study employed the CellSearch test (Janssen Diagnostics), an EpCAM-based, FDA-cleared blood test for CTC enumeration. CTC counts closely correlated with tumor load, and in patients with distant metastases, median overall survival of 2 and 1 months was reported for patients with a count of CTCs ≥2 or ≥5/ blood sample, respectively. The prognostic impact of baseline CTC status and overall survival was also confirmed in 95 patients with advanced BTC included in the Advanced Biliary tract Cancer (ABC)-03 trial, with a 1-CTC positivity cutoff [118]. CTCs might therefore be a valuable tool to guide treatment strategies in the adjuvant and palliative setting.

Beyond their prognostic value, isolated CTC can be used for molecular characterization of the tumor and provide the opportunity to explore intra-patient tumor heterogeneity and potential target therapies. In this sense, CTCs were isolated from blood samples obtained by intraoperative venipuncture during pancreaticoduodenectomy by fluorescent-activated cell sorting (CD44^+^, CD147^+^, EpCAM^+^ and CD45^−^) in which *KRAS* mutations were detected [119]. Noteworthy, mutant *KRAS* CTCs were later shown to be highly proliferative, resistant to apoptotic cell death and also able to recruit multiple cells from the immune system [120]. These findings thus indicate that CTCs survival within the portal vein might interact with multiple cell types, possibly representing a source of local recurrence and metastasis. Additionally, protocols have been developed to culture CTCs for extended periods of time in vitro, especially as 3D organoid cultures [121]. These cells, either derived from CTCs or facilitate not only in vitro drug screenings, but may also be expanded in immunocompromised mice to test for treatment sensitivity in vivo, thus moving a step forward in personalized medicine and allowing the selection of the most suitable therapeutic regimens [122].

## 4. Biomarkers in Tumor Tissue

Tumor tissue biomarkers embody not only diagnostic value but might also may aid to predict prognosis (overall survival and/or tumor recurrence) or even guide therapeutic decisions and assess responses to potential adjuvant therapies. Although a high genomic heterogeneity is evident in CCA tumors, specific genetic alterations were reported according to the cancer subtype. For instance, while small bile duct iCCAs usually present mutations in *IDH1/2* and *FGFR2* fusions, large duct iCCAs, pCCAs and dCCAs more constantly display *KRAS* and/or *TP53* mutations. Interestingly, *ELF3* mutations seem to be exclusively found in patients with dCCA. Genomic and transcriptomic studies have been able to also identify specific signatures related with tumor development and progression. In this setting, the most prevalent alterations reported in CCA were related with cell proliferation (*KRAS*, *BRAF*, *SMAD4*, *FGFR2* and *PTPN3*) [123,124,125,126,127,128,129], developmental pathways linked to cancer growth (*NOTCH1*, *NICD*, *WNT7B* and *WNT10A*) [130,131], DNA repair (*TP53*) [123,124,125,132,133], and chromatin remodeling (*KMT2C*, *ARID1A*, *PBRM1* and *BAP1*) [132,133] Furthermore, transcriptomic analysis of iCCA tumors allowed the identification of two distinct subtypes: the “inflammation subtype”, mainly enriched in inflammatory-related genes and the “proliferation subtype”, which was characterized by the increased expression of oncogenes and related with worse prognosis [128], pinpointing the relevance of tissue biomarkers in predicting prognosis.

Similarly, mRNA microarray analysis conducted in tumor samples from surgically resected iCCAs resulted in the description of a 36-gene panel correlated with worse disease outcome (overall survival). *KRAS*/*BRAF* mutations were also directly linked to poor prognosis, in parallel with increased expression of Erb-B2 Receptor Tyrosine Kinase 2 (HER2), which was not detected in samples obtained from patients with a good prognosis [134]. The prognostic value of mutational genomic analysis was later corroborated in two independent international studies that reported a worse overall survival and higher recurrence in patients with iCCA harboring *KRAS* (12–16%) and *TP53* (13–20%) mutations, when compared with patients with genetic alterations in *IDH1/2* gene or with the undetermined group (including none of the previously mentioned mutations) [127,135]. Furthermore, specific mutational fingerprints might be more prevalent in specific CCA subtypes or associated with particular etiological and/or risk factors. Mutations on *TP53* gene are highly frequent in patients with chronic HBV infection. Furthermore, genome sequencing of liver fluke (*O. viverrini* and *C. sinensis*)-positive tumors revealed a higher mutational rate and increased prevalence of *SMAD4* and *TP53* mutations, in parallel with ERBB2 amplifications, when compared with liver fluke-negative tumors [29,123,133]. In this sense, genome wide-association studies (GWAS) will provide more advances in this field in a near future.

*FGFR2* gene fusions [125,127,136], *IDH1/2* mutations [125,126,132,133,137,138] and neurotropic tyrosine kinase receptor (*NTKR*) fusions [125] are mostly exclusively found in patients with iCCA (5.5–13.6%, 4.9–36% and 3.5%, respectively), and are currently under the spotlight in the drug discovery thematic considering their potential therapeutic targeting. In this sense, four phase II clinical trials have already described the benefit of FGFR2-directed therapies in patients with iCCA harboring FGFR2 alterations who progressed under first line treatment with the standard of care (gemcitabine-cisplatin), reporting promising results. Noteworthy, the FDA- and EMA-approved FGFR inhibitor pemigatinib showed an objective response of 35.5%, resulting in a median progression-free survival of 6.9 months in the FIGHT-202 trial [36]. The use of this and other FGFR inhibitors as a first-line treatment, compared to the standard of care, in patients with advanced CCA is now currently being evaluated (NCT03656536, NCT03773302, NCT04093362). Similarly, the therapeutic efficacy of the IDH inhibitors (NTC02428855; NTC02989857; NTC02381886) is currently being tested for patients with IDH-mutant iCCA and, in parallel, targeting NTRK fusions with larotrectinib [139] or entrectinib [140] have shown promising results for the treatment of previously treated advanced solid tumors, including also CCA. In addition, the presence of mismatch repair (MMR) deficiency and/or microsatellite instability (MSI) might be a good indicator of response to immunotherapies since MSI tumors harbor an increased number of genetic mutations and MMR deficiency and/or MSI usually presented an increased number of neoepitopes and CD8^+^ T cell infiltration and an improved response to anti-PDL1 monoclonal antibodies [141].

Several tissue biomarkers were reported to predict disease prognosis and recurrence [13]. A meta-analysis including 73 immunohistochemistry-based studies including 4126 patients with CCA revealed the prognostic value of 77 proteins in patients undergoing surgical resection. Among them, fascin, epidermal growth factor receptor (EGFR), p27, mucin 1 (MUC1) and MUC4 were identified as independent prognostic factors, associated with worse overall survival [142]. In parallel, 39 transcriptomic-based, immune response-related prognostic biomarkers were reported in 53 patients with BTC whom underwent tumor resection, with cytotoxic T-lymphocyte antigen 4 (CTL4) correlating with recurrence-free survival, while the levels of CD80 did not predict recurrence per se but allowed to predict prognosis in patients receiving adjuvant chemotherapy [143]. Additionally, the high tumor tissue levels of IL-33 correlated with a favorable prognosis in patients with either iCCA or pCCA [144] while granulocyte colony-stimulating factor (G-CSF) were suggested as a prognostic biomarker to predict tumor recurrence after resection [145,146].

The aberrant expression of non-coding RNAs in tumor CCA tissues was already reported and may also possess prognostic value [147,148]. Among them, regardless of its great diagnostic capacity, the oncomiR-21 is rapidly arising as a promising prognostic biomarker since its expression in tumor tissue positively correlated with clinical stage at diagnosis, tumor differentiation status and also with overall and progression-free survival [49,149]. Similarly, miR-383 was shown to be highly upregulated in patients with CCA, promoting tumor cell proliferation, migration, and invasion in an interferon regulatory factor-1 (IRF1)-dependent manner and positively correlating with advanced tumor stage, large tumor size, invasion, and metastasis, and being regarded as an unfavorable independent prognostic factor [150]. Finally, miRNA tissue panels might also aid in the diagnosis of CCA since a panel of 7 miRNAs was reported to allow the differential diagnosis of tumors with similar clinical presentations, such as CCA and pancreatic adenocarcinoma [151] but the prognostic value of this type of panels is yet to be unveiled.

## 5. Conclusions and Future Perspectives

**Figure 1:**
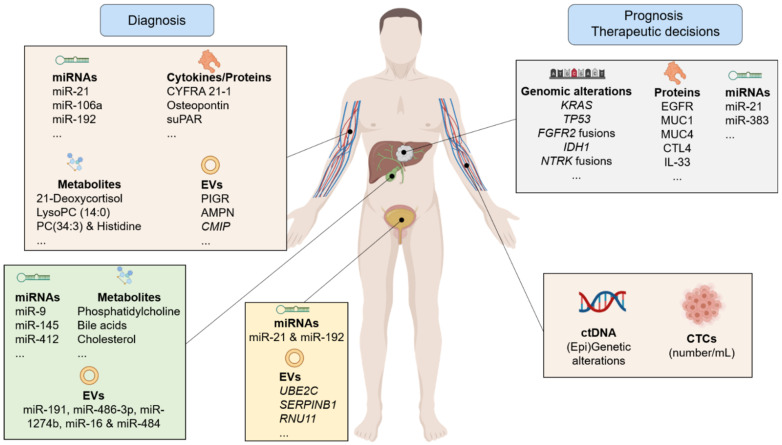
Next-generation biomarkers for cholangiocarcinoma. Created with biorender.com.

The use of the single term “cholangiocarcinoma” belies the complexity and heterogeneity of a diverse collection of malignancies arising within the biliary tract. To date, classification has largely relied on the anatomical site of origin coupled with histopathological morphology and immunohistochemistry. An increasing understanding of the biology of CCA based on next-generation sequencing of tumor tissue has identified a number of molecular subgroups harboring actionable mutations, with the accelerating emergence of novel treatment options (FGFR2 fusion, NTRK fusion and IDH1 inhibitors, for example with many others under evaluation). Still, regarding diagnosis, the limitations of tissue acquisition are well-recognized and the emergence of blood-based circulating biomarkers (ranging from whole CTCs, to ctDNA, miRNAs, EVs, cytokines, proteins and metabolites) is providing numerous platforms for investigation. Particularly, the identification of early diagnostic biomarkers will result in an increase in the number of patients who might benefit from potentially curative approaches, such as surgical tumor resection and/or liver transplantation. The ability to evaluate these biomarkers in bile and urine as well as blood, provides an opportunity to maximize the information that can be obtained from an individual patient. Furthermore, the high number of novel biomarkers recently proposed (Table 1, Table 2, Table 3 and Table 4; Figure 1) clearly highlight CCA tumor heterogeneity and mirror the difficulty to find robust non-invasive biomarkers. Most of the published reports arise from early exploratory studies that deserve further validation studies and consequently, each biomarker, alone or as part of a panel, needs now a careful evaluation within a specific pre-defined purpose (diagnostic, prognostic, predictive of efficacy to therapy, predictive of toxicity, pharmacodynamic, detection of primary or acquired resistance) with subsequent validation in adequately powered studies and using easily transferable techniques that might be used in clinical daily routine. In this regard, conducting large international validation studies, including well-defined and appropriate control groups and patients with biopsy-proven CCA from different subtypes and also specific subgroups known to predispose to CCA development is eagerly awaited.

Due to the infrequency of CCA, international collaborative studies are essential and in addition to their role within established anatomical and molecular subgroups, the role of biomarkers in CCA arising within different global regions with different environmental risks (e.g., viral hepatitis, metabolic syndrome, liver fluke exposure, etc.) is also warranted. The H2020 ESCALON project is based on a European–Latin American Consortium that aims to describe and validate accurate biomarkers for the diagnosis of liver cancer, including CCA, not only in Europe and South America, but also in other continents, which will certainly help to fill in this gap. Finally, the accurate discovery and validation of accurate biomarkers relies on the close collaboration of dedicated Centres in the field and for instance, the efforts being employed by the European Network for the Study of Cholangiocarcinoma (ENS-CCA: http://www.enscca.org/www.cholangiocarcinoma.eu) (accessed on 16 May 2021), a pan-European and multidisciplinary collaborative network, and by the EURO-CHOLANGIONET COST Action have been driven in this direction, constituting the best platform to engage and conduct these validation studies. Overall, although we still lack accurate non-invasive biomarkers to either identify CCA or predict disease progression and prognosis, we are currently in the good way to describe next-generation biomarkers that, after proper validation, will be translated into clinics, ultimately improving patients’ welfare and improving the prognosis of this devastating cancer.

## Data Availability

No new data were created or analyzed in this study. Data sharing is not applicable to this article.
